# Some means inequalities for positive operators in Hilbert spaces

**DOI:** 10.1186/s13660-016-1283-x

**Published:** 2017-01-10

**Authors:** Jin Liang, Guanghua Shi

**Affiliations:** School of Mathematical Sciences, Shanghai Jiao Tong University, Shanghai, 200240 China

**Keywords:** 47A30, 47A63, 26D10, 26D20, 26E60, means inequalities, positive linear operators, Hilbert space

## Abstract

In this paper, we obtain two refinements of the ordering relations among Heinz means with different parameters via the Taylor series of some hyperbolic functions and by the way, we derive new generalizations of Heinz operator inequalities. Moreover, we establish a matrix version of Heinz inequality for the Hilbert-Schmidt norm. Finally, we introduce a weighted multivariate geometric mean and show that the weighted multivariate operator geometric mean possess several attractive properties and means inequalities.

## Introduction

Since Heinz proved a series of useful norm inequalities, which are closely related to the Cordes inequality and the Furuta inequality, in 1951, many researchers have devoted themselves to sharping the Heinz inequalities and extending the Heinz norm inequalities to more general cases with the help of a Bernstein type inequality for nonselfadjoint operators, the convexity of norm functions, the Jensen functional and its properties, the Hermite-Hadamard inequality, and so on. With this kind of research, the study of various means inequalities, such as the geometric mean, the arithmetic mean, the Heinz mean, arithmetic-geometric means, and Arithmetic-Geometric-Harmonic (A-G-H) weighted means, has received much attention and development too. For recent interesting work in this area, we refer the reader to [1–10] and references therein.

Based on [1–8], in this paper, we are concerned with the further refinements of the geometric mean and the Heinz mean for operators in Hilbert spaces. Our purpose is to derive some new generalizations of Heinz operator inequalities by refining the ordering relations among Heinz means with different parameters, and of the geometric mean by investigating geometric means of several operator variables in a weighted setting. Moreover, we will obtain a matrix version of the Heinz inequality for the Hilbert-Schmidt norm.

Throughout this paper, $\mathfrak{B}^{++}(\mathcal{H})$ stands for the set of all bounded positive invertible operators on a Hilbert space $\mathcal{H}$, $\mathfrak{B}(\mathcal{H})$ is the set of all bounded linear operators on $\mathcal{H}$, and $\mathfrak{B}(\mathcal{H})_{\mathrm{sa}}$ is a convex domain of selfadjoint operators in $\mathfrak{B}(\mathcal {H})$. For any $T,S\in\mathfrak{B}^{++}(\mathcal{H}) $ and $\nu\in [0,1]$, we write

$$\begin{aligned} T\nabla_{\nu}S:=(1-\nu)T+\nu S \end{aligned}$$

and

$$\begin{aligned} T\sharp_{\nu}S:=T^{\frac{1}{2}}\bigl(T^{-\frac {1}{2}}ST^{-\frac{1}{2}} \bigr)^{\nu}T^{\frac{1}{2}} \end{aligned}$$

as the geometric mean of *T* and *S*. When $\nu=1/2$ we write $T\nabla S$ and $T\sharp S$ in short, respectively. We refer the reader to Kubo and Ando [6] for more information on the means of positive linear operators.

Recall that, for any $a,b\geq0$, the number

$$\begin{aligned} H_{\nu}(a,b)=\frac{a^{1-\nu}b^{\nu}+a^{\nu}b^{1-\nu }}{2},\quad0\leq\nu\leq1, \end{aligned}$$

is called the Heinz mean $H_{\nu}(a,b)$ of *a* and *b*. It is clear that

$$\begin{aligned} &{H_{0}(a,b)=H_{1}(a,b)=\frac{a+b}{2}, \qquad H_{1/2}(a,b)=\sqrt{ab},} \\ &{H_{\nu}(a,b)=H_{1-\nu}(a,b),\quad\nu\in[0,1],} \\ &{\sqrt{ab}\leq H_{\nu}(a,b)\leq\frac{a+b}{2}, \quad\mbox{$\nu \in[0,1]$}.} \end{aligned}$$

For any $T,S\in\mathfrak{B}^{++}(\mathcal{H})$ and $\nu\in[0,1]$, the operator

$$\begin{aligned} H_{\nu}(T,S)=\frac{T\sharp_{\nu}S+T\sharp_{1-\nu}S}{2} \end{aligned}$$

is called the Heinz operator mean of *T* and *S*. Clearly,

$$\begin{aligned} T\sharp S\leq H_{\nu}(T,S)\leq T\nabla S, \end{aligned}$$

that is, the Heinz operator mean interpolates between the geometric mean and the arithmetic mean.

## Improved Heinz means inequalities

In a very recent work [8], we establish the following inequalities:

2.1 $$\begin{aligned} H_{t}(a,b)\leq \biggl(1-\frac{(1-2t)^{2}}{(1-2s)^{2}} \biggr)a^{\frac{1}{2}}b^{\frac{1}{2}}+ \frac{(1-2t)^{2}}{(1-2s)^{2}}H_{s}(a,b) \end{aligned}$$

and

$$\begin{aligned} H_{t}(T,S)\leq \biggl(1-\frac{(1-2t)^{2}}{(1-2s)^{2}} \biggr)T\sharp S+ \frac{(1-2t)^{2}}{(1-2s)^{2}}H_{s}(T,S) \end{aligned}$$

hold for $s,t\in[0,1]$ satisfying $\vert s-\frac{1}{2}\vert \geq \vert t-\frac {1}{2}\vert $, $s\neq\frac{1}{2}$.

In this section, we improve the result and give two theorems as follows.

### Theorem 2.1

*Suppose* $T,S\in\mathfrak{B}^{++}(\mathcal{H})$, *and let* $s,t\in[0,1]$ *satisfy*

$$s\neq\frac{1}{2},\qquad\biggl\vert s-\frac{1}{2}\biggr\vert \geq \biggl\vert t-\frac{1}{2}\biggr\vert . $$

*Then*

2.2 $$\begin{aligned} &{\biggl(1-\frac{(1-2t)^{2}}{(1-2s)^{2}} \biggr)T\sharp S+\frac{(1-2t)^{2}}{(1-2s)^{2}}H_{s}(T,S)} \\ &{\quad \leq \bigl(1-{(1-2t)^{2}}\bigr)T\sharp S+{(1-2t)^{2}}T \nabla S.} \end{aligned}$$

### Proof

Writing

$$\alpha=1-2s, \qquad\beta=1-2t, $$

we see

$$\alpha\neq0,\quad0\leq \vert \beta \vert \leq \vert \alpha \vert \leq1. $$

In view of the Taylor series of cosh*x*, we deduce that

$$\begin{aligned} &{\bigl(1-\beta^{2}\bigr)+{\beta^{2}}\cosh x- \biggl[ \biggl(1-\frac {\beta^{2}}{\alpha^{2}} \biggr)+\frac{\beta^{2}}{\alpha^{2}}\cosh\alpha x \biggr]} \\ &{\quad = \frac{\beta^{2}}{\alpha^{2}} \bigl(1-\alpha^{2}+\alpha^{2}\cosh x- \cosh\alpha x \bigr)} \\ &{\quad = \frac{\beta^{2}}{\alpha^{2}} \biggl[1-\alpha^{2}+ \biggl(\alpha^{2}+ \frac{\alpha^{2} x^{2}}{2!}+\frac{\alpha^{2}x^{4}}{4!}+\frac{\alpha^{2}x^{6}}{6!}+\cdots \biggr)} \\ &{\qquad{} - \biggl(1+\frac{\alpha^{2} x^{2}}{2!}+\frac{\alpha^{4}x^{4}}{4!}+\frac{\alpha ^{6}x^{6}}{6!}+\cdots \biggr) \biggr]} \\ &{\quad = \frac{\beta^{2}}{\alpha^{2}} \biggl[ \biggl(\frac{\alpha^{2}x^{4}}{4!}+\frac{\alpha ^{2}x^{6}}{6!}+\cdots \biggr)- \biggl(\frac{\alpha^{4}x^{4}}{4!}+\frac{\alpha ^{6}x^{6}}{6!}+\cdots \biggr) \biggr]} \\ &{\quad \geq 0,} \end{aligned}$$

i.e.,

$$\begin{aligned} \biggl(1-\frac{\beta^{2}}{\alpha^{2}} \biggr)+\frac{\beta ^{2}}{\alpha^{2}}\cosh\alpha x \leq& \bigl(1-\beta^{2}\bigr)+{\beta^{2}}\cosh x. \end{aligned}$$

With $\frac{x-y}{2}$ instead of *x*, we have

$$\begin{aligned} &{ \biggl(1-\frac{(1-2t)^{2}}{(1-2s)^{2}} \biggr)+\frac {(1-2t)^{2}}{(1-2s)^{2}}\cosh \biggl((1-2s) \frac{x-y}{2} \biggr)} \\ &{\quad \leq \bigl(1-(1-2t)^{2}\bigr)+{(1-2t)^{2}}\cosh \biggl( \frac{x-y}{2} \biggr).} \end{aligned}$$

Let $a=e^{x}$, $b=e^{y}$. Then

2.3 $$\begin{aligned} &{\biggl(1-\frac{(1-2t)^{2}}{(1-2s)^{2}} \biggr)\sqrt{ab}+\frac {(1-2t)^{2}}{(1-2s)^{2}}H_{s}(a,b)} \\ &{\quad \leq \bigl(1-(1-2t)^{2}\bigr)\sqrt {ab}+{(1-2t)^{2}} \frac{a+b}{2}.} \end{aligned}$$

Taking $a=x$ and $b=1$ in the inequality (2.2), we get

$$\begin{aligned} \biggl(1-\frac{(1-2t)^{2}}{(1-2s)^{2}} \biggr)x^{1/2}+\frac {(1-2t)^{2}}{(1-2s)^{2}} \biggl( \frac{x^{1-s}+x^{s}}{2} \biggr) \leq& \bigl(1-(1-2t)^{2}\bigr)x^{1/2}+{(1-2t)^{2}} \frac{x+1}{2}. \end{aligned}$$

With the positive operator $T^{-\frac{1}{2}}ST^{-\frac{1}{2}}$ instead of *x*, we have

$$\begin{aligned} \biggl(1-\frac{(1-2t)^{2}}{(1-2s)^{2}} \biggr)T\sharp S+\frac {(1-2t)^{2}}{(1-2s)^{2}}H_{s}(T,S) \leq& \bigl(1-{(1-2t)^{2}}\bigr)T\sharp S+{(1-2t)^{2}}T\nabla S. \end{aligned}$$

The proof is completed. □

For the functions $F_{\nu}:\mathbb{R}_{+}\rightarrow\mathbb{R}$ $(\nu\in [0,1])$ defined by

$$\begin{aligned} F_{\nu}(x)= \textstyle\begin{cases} \frac{x^{\nu}-x^{1-\nu}}{\log x}, &{x>0, x\neq1,}\\ 2\nu-1, & {x=1,} \end{cases}\displaystyle \end{aligned}$$

we have the following result.

### Theorem 2.2

*Suppose* $T,S\in\mathfrak{B}^{++}(\mathcal{H})$ *and let* $s,t\in[0,1]$ *satisfy*

$$s,t\neq\frac{1}{2},\qquad\biggl\vert s-\frac{1}{2}\biggr\vert \geq\biggl\vert t-\frac{1}{2}\biggr\vert . $$

*Then*

$$\begin{aligned} &{\frac{1}{2t-1}T^{\frac{1}{2}}F_{t}\bigl(T^{-\frac {1}{2}}ST^{-\frac{1}{2}} \bigr)T^{\frac{1}{2}}} \\ &{\quad\leq \biggl(1-\frac {(1-2t)^{2}}{(1-2s)^{2}} \biggr)T\sharp S+ \frac{(1-2t)^{2}}{(1-2s)^{2}}\frac {1}{2s-1}T^{\frac{1}{2}}F_{s} \bigl(T^{-\frac{1}{2}}ST^{-\frac{1}{2}}\bigr)T^{\frac{1}{2}}.} \end{aligned}$$

### Proof

Writing $\alpha=1-2s$, $\beta=1-2t$, we have

$$\alpha\neq0,\qquad\beta\neq0, \qquad0\leq \vert \beta \vert \leq \vert \alpha \vert \leq1. $$

It follows from the Taylor series of sinh*x* that

$$\begin{aligned} \frac{\sinh\beta x}{\beta x} =&1+\frac{\beta^{2}x^{2}}{3!}+\frac{\beta^{4}x^{4}}{5!}+\frac{\beta ^{6}x^{6}}{7!}+ \cdots \\ \leq& 1-\frac{\beta^{2}}{\alpha^{2}}+\frac{\beta^{2}}{\alpha^{2}} +\frac {\beta^{2}}{\alpha^{2}} \biggl( \frac{\alpha^{2}x^{2}}{3!}+\frac{\alpha ^{4}x^{4}}{5!}+\frac{\alpha^{6}x^{6}}{7!}+\cdots \biggr) \\ \leq& 1-\frac{\beta^{2}}{\alpha^{2}}+\frac{\beta^{2}}{\alpha^{2}} \biggl(1+ \frac{\alpha^{2}x^{2}}{3!}+ \frac{\alpha^{4}x^{4}}{5!}+\frac{\alpha ^{6}x^{6}}{7!}+\cdots \biggr) \\ =& \biggl(1-\frac{\beta^{2}}{\alpha^{2}} \biggr)+\frac{\beta^{2}}{\alpha^{2}} \biggl( \frac{\sinh\alpha x}{\alpha x} \biggr). \end{aligned}$$

With $\frac{x-y}{2}$ instead of *x*, we know that, for any $s,t\in [0,1]$ satisfying $\vert s-\frac{1}{2}\vert \geq \vert t-\frac{1}{2}\vert $, $s, t \neq\frac{1}{2}$,

$$\begin{aligned} \frac{\sinh ( (1-2t) (\frac{x-y}{2} ) )}{(1-2t) (\frac{x-y}{2} )}\leq \biggl(1-\frac {(1-2t)^{2}}{(1-2s)^{2}} \biggr)+\frac{(1-2t)^{2}}{(1-2s)^{2}} \biggl(\frac{\sinh ((1-2s) (\frac{x-y}{2} ) )}{(1-2s) (\frac {x-y}{2} )} \biggr). \end{aligned}$$

Put $a=e^{x}$, $b=e^{y}$. Then we get

2.4 $$\begin{aligned} &{\frac{a^{1-t}b^{t}-a^{t}b^{1-t}}{(1-2t)(\log a-\log b)}} \\ &{\quad \leq \biggl(1-\frac{(1-2t)^{2}}{(1-2s)^{2}} \biggr)a^{\frac{1}{2}}b^{\frac {1}{2}}+ \frac{(1-2t)^{2}}{(1-2s)^{2}} \biggl(\frac {a^{1-t}b^{t}-a^{t}b^{1-t}}{(1-2t)(\log a-\log b)} \biggr).} \end{aligned}$$

Letting $a=x$ and $b=1$ in inequality (2.3), we see that

$$\begin{aligned} \frac{x^{t}-x^{1-t}}{(2t-1)\log x}\leq \biggl(1-\frac {(1-2t)^{2}}{(1-2s)^{2}} \biggr)x^{\frac{1}{2}}+ \frac{(1-2t)^{2}}{(1-2s)^{2}} \biggl(\frac{x^{t}-x^{1-t}}{(2t-1)\log x} \biggr), \end{aligned}$$

that is,

$$\begin{aligned} \frac{1}{2t-1}F_{t}(x)\leq \biggl(1-\frac {(1-2t)^{2}}{(1-2s)^{2}} \biggr)x^{\frac{1}{2}}+\frac{(1-2t)^{2}}{(1-2s)^{2}}\frac {1}{2s-1}F_{s}(x). \end{aligned}$$

With the positive operator $T^{-\frac{1}{2}}ST^{-\frac{1}{2}}$ instead of *x*, we have

$$\begin{aligned} &{ \frac{1}{2t-1}F_{t}\bigl(T^{-\frac{1}{2}}ST^{-\frac {1}{2}}\bigr)} \\ &{\quad \leq \biggl(1-\frac{(1-2t)^{2}}{(1-2s)^{2}} \biggr) \bigl(T^{-\frac {1}{2}}ST^{-\frac{1}{2}} \bigr)^{\frac{1}{2}} +\frac{(1-2t)^{2}}{(1-2s)^{2}}\frac{1}{2s-1}F_{s} \bigl(T^{-\frac{1}{2}}ST^{-\frac{1}{2}}\bigr).} \end{aligned}$$

Therefore,

$$\begin{aligned} &{\frac{1}{2t-1}T^{\frac{1}{2}}F_{t}\bigl(T^{-\frac {1}{2}}ST^{-\frac{1}{2}} \bigr)T^{\frac{1}{2}}} \\ &{\quad \leq \biggl(1-\frac {(1-2t)^{2}}{(1-2s)^{2}} \biggr)T\sharp S + \frac{(1-2t)^{2}}{(1-2s)^{2}}\frac{1}{2s-1}T^{\frac{1}{2}}F_{s} \bigl(T^{-\frac {1}{2}}ST^{-\frac{1}{2}}\bigr)T^{\frac{1}{2}}.} \end{aligned}$$

 □

## Heinz inequality for the Hilbert-Schmidt norm

In this section, we let $M_{n}$ be the Hilbert space of $n\times n$ complex matrices and let $\Vert \cdot \Vert $ stand for any unitarily invariant norm on $M_{n}$, i.e. $\Vert UTV\Vert =\Vert T\Vert $ for all $T\in M_{n}$ and for all unitary matrices $U,V\in M_{n}$. We suppose that $T, S, X \in M_{n}$ with *T* and *S* being positive semidefinite. For $T=[a_{ij}]\in M_{n}$, the Hilbert-Schmidt norm of *T* is defined by

$$\Vert T\Vert _{2}= \Biggl(\sum_{i,j=1}^{n}|a_{ij}| ^{2} \Biggr)^{{1}/{2}}. $$

It is well known that the Hilbert-Schmidt norm is unitarily invariant.

Next, we prove the following matrix version of Heinz inequality for the Hilbert-Schmidt norm.

### Theorem 3.1

*Let* $s,t\in[0,1]$ *satisfy*

$$s\neq\frac{1}{2},\qquad\biggl\vert s-\frac{1}{2}\biggr\vert \geq \biggl\vert t-\frac{1}{2}\biggr\vert . $$

*Then*

$$\begin{aligned} &{ \biggl\Vert \frac{T^{t}XS^{1-t}+T^{1-t}XS^{t}}{2}\biggr\Vert _{2}} \\ &{\quad \leq \biggl\Vert \biggl(1-\frac{(1-2t)^{2}}{(1-2s)^{2}} \biggr)T^{1/2}XS^{1/2}+ \frac{(1-2t)^{2}}{(1-2s)^{2}}\frac {T^{s}XS^{1-s}+T^{1-s}XS^{s}}{2}\biggr\Vert _{2}} \\ &{\quad \leq \biggl\Vert \bigl(1-{(1-2t)^{2}}\bigr)T^{1/2}XS^{1/2}+(1-2t)^{2} \frac{TX+XS}{2}\biggr\Vert _{2}.} \end{aligned}$$

### Proof

Noting that *T* and *S* are positive semidefinite, we know by the spectral theorem that there exist unitary matrices $U, V \in M_{n}$ such that

$$T=U\Lambda_{1}U^{*}\quad\mbox{and}\quad S=V\Lambda_{2}V^{*}, $$

where

$$\Lambda_{1}=\operatorname{diag}(\lambda_{1}, \ldots, \lambda_{n}), \qquad\Lambda_{2}=\operatorname{diag}(\mu _{1}, \ldots, \mu_{n}), \quad\lambda_{i}, \mu_{i}\geq0, i=1, \ldots, n. $$

Put

$$Y=U^{*}XV=[y_{ij}]. $$

Then we have

$$\begin{aligned} \frac{T^{t}XS^{1-t}+T^{1-t}XS^{t}}{2} =& \frac{(U\Lambda_{1}U^{*})^{t}X(V\Lambda_{2}V^{*})^{1-t}+(U\Lambda _{1}U^{*})^{1-t}X(V\Lambda_{2}V^{*})^{t}}{2} \\ =& \frac{(U\Lambda_{1}^{t}U^{*})X(V\Lambda_{2}^{1-t}V^{*})+(U\Lambda _{1}^{1-t}U^{*})X(V\Lambda_{2}^{t}V^{*})}{2} \\ =& \frac{U\Lambda_{1}^{t}(U^{*}XV)\Lambda_{2}^{1-t}V^{*}+U\Lambda _{1}^{1-t}(U^{*}XV)\Lambda_{2}^{t}V^{*}}{2} \\ =& U \biggl(\frac{\Lambda_{1}^{t}Y\Lambda_{2}^{1-t}+\Lambda_{1}^{1-t}Y\Lambda _{2}^{t}}{2} \biggr)V^{*}. \end{aligned}$$

Hence,

$$\begin{aligned} \biggl\Vert \frac{T^{t}XS^{1-t}+T^{1-t}XS^{t}}{2}\biggr\Vert _{2}^{2} =& \biggl\Vert \frac{\Lambda_{1}^{t}Y\Lambda_{2}^{1-t}+\Lambda_{1}^{1-t}Y\Lambda _{2}^{t}}{2}\biggr\Vert _{2}^{2} \\ =& \sum_{i,j=1}^{n} \biggl(\frac{\lambda_{i}^{t}\mu_{j}^{1-t}+\lambda_{i}^{1-t}\mu _{j}^{t}}{2} \biggr)^{2}\vert y_{ij}\vert ^{2}. \end{aligned}$$

By a similar argument to the above, we deduce that

$$\begin{aligned} &{\biggl\Vert \biggl(1-\frac{(1-2t)^{2}}{(1-2s)^{2}} \biggr)T^{1/2}XS^{1/2}+ \frac{(1-2t)^{2}}{(1-2s)^{2}}\frac{T^{s}XS^{1-s}+T^{1-s}XS^{s}}{2}\biggr\Vert _{2}^{2}} \\ &{\quad = \sum_{i,j=1}^{n} \biggl( \biggl(1- \frac{(1-2t)^{2}}{(1-2s)^{2}} \biggr)\sqrt {\lambda_{i}\mu_{j}}+ \biggl( \frac{(1-2t)^{2}}{(1-2s)^{2}} \biggr)\frac{\lambda_{i}^{s}\mu _{j}^{1-s}+\lambda_{i}^{1-s}\mu_{j}^{s}}{2} \biggr)^{2}\vert y_{ij}\vert ^{2}} \end{aligned}$$

and

$$\begin{aligned} &{\biggl\Vert \bigl(1-{(1-2t)^{2}}\bigr)T^{1/2}XS^{1/2}+(1-2t)^{2} \frac {TX+XS}{2}\biggr\Vert _{2}^{2}} \\ &{\quad = \sum_{i,j=1}^{n} \biggl( \bigl(1-{(1-2t)^{2}}\bigr)\sqrt{\lambda_{i} \mu_{j}} +{(1-2t)^{2}}\frac{\lambda_{i}+\mu_{j}}{2} \biggr)^{2}\vert y_{ij}\vert ^{2}.} \end{aligned}$$

By virtue of the inequalities (2.1) and (2.2), we obtain

$$\begin{aligned} &{\sum_{i,j=1}^{n} \biggl(\frac{\lambda_{i}^{t}\mu _{j}^{1-t}+\lambda_{i}^{1-t}\mu_{j}^{t}}{2} \biggr)^{2}\vert y_{ij}\vert ^{2}} \\ &{\quad \leq \sum_{i,j=1}^{n} \biggl( \biggl(1- \frac{(1-2t)^{2}}{(1-2s)^{2}} \biggr)\sqrt {\lambda_{i}\mu_{j}}+ \biggl( \frac{(1-2t)^{2}}{(1-2s)^{2}} \biggr)\frac{\lambda_{i}^{s}\mu _{j}^{1-s}+\lambda_{i}^{1-s}\mu_{j}^{s}}{2} \biggr)^{2}\vert y_{ij}\vert ^{2}} \\ &{\quad \leq \sum_{i,j=1}^{n} \biggl( \bigl(1-{(1-2t)^{2}}\bigr)\sqrt{\lambda_{i} \mu_{j}} +{(1-2t)^{2}}\frac{\lambda_{i}+\mu_{j}}{2} \biggr)^{2}\vert y_{ij}\vert ^{2}.} \end{aligned}$$

Thus, the proof is completed. □

## The inductive weighted geometric means and means inequalities

Let $F: \mathcal{D}\rightarrow\mathfrak{B}(\mathcal{H})$ be a mapping of *k* variables defined in a convex domain $\mathcal{D}\subseteq \mathfrak{B}(\mathcal{H})^{k}$. Recall from Hansen [7] that *F* is regular if:

(i) The domain $\mathcal{D}$ is invariant under unitary transformations of $\mathcal{H}$ and

$$F\bigl(U^{*} T_{1} U,\ldots, U^{*} T_{k} U\bigr)=U^{*}F(T_{1}, \ldots, T_{k})U $$

for every $(T_{1},\ldots, T_{k})\in\mathcal{D}$ and every unitary *U* on $\mathcal{H}$.

(ii) Let *P* and *Q* be mutually orthogonal projections acting on $\mathcal{H}$ and take arbitrary *k*-tuples $(T_{1},\ldots ,T_{k})$ and $(S_{1},\ldots,S_{k})$ of operators in $\mathfrak{B}(\mathcal{H})$ such that the compressed tuples $(PT_{1}P,\ldots,PT_{k}P)$ and $(QS_{1}Q, \ldots, QS_{k}Q)$ are in the domain $\mathcal{D}$. Then the *k*-tuple of diagonal block matrices

$$(PT_{1}P+QS_{1}Q,\ldots,PT_{k}P+QS_{k}Q) $$

is also in the domain $\mathcal{D}$ and

$$\begin{aligned} &{F(PT_{1}P+QS_{1}Q,\ldots,PT_{k}P+QS_{k}Q)} \\ &{\quad = PF(PT_{1}P,\ldots ,PT_{k}P)P+QF(QS_{1}Q, \ldots,QS_{k}Q)Q.} \end{aligned}$$

Recall also from Hansen [7] that the perspective of a regular operator mapping of several variables is defined as

$$\mathcal{P}_{F}(T_{1},\ldots, T_{k}, S)=S^{1/2}{F}\bigl(S^{-1/2}T_{1}S^{-1/2}, \ldots,S^{-1/2}T_{k}S^{-1/2}\bigr)S^{1/2}. $$

Hansen [7] proves that the perspective $\mathcal{P}_{F}$ of a convex regular map $F:\mathcal{D}^{k}_{+}\rightarrow\mathfrak{B}(\mathcal {H})$ is regular and convex.

Write

$$\mathcal{D}^{k}_{+} =\bigl\{ (T_{1},\ldots, T_{k}) | T_{1},\ldots, T_{k}> 0\bigr\} . $$

Now we prove another two properties of $\mathcal{P}_{F}$.

### Theorem 4.1

*Suppose that* $F: \mathcal{D}^{k}_{+}\rightarrow\mathfrak {B}(\mathcal{H})_{\mathrm{sa}}$ *is regular*, *concave*, *and continuous*. *Then the perspective function* $\mathcal{P}_{F}$ *is monotone*.

### Proof

Let $T_{i}$ and $S_{i}$ be positive invertible operators such that $T_{i}\leq S_{i}$ for $i=1,\ldots, k+1$. If $S_{i}-T_{i}$ is invertible for each $i=1,\ldots, k+1$ and $\lambda\in(0,1)$, then we have

$$\lambda S_{i}=\lambda T_{i}+(1-\lambda)W_{i}, \quad i=1,\ldots, k+1, $$

where

$$W_{i}=\lambda(1-\lambda)^{-1}(S_{i}-T_{i}), \quad i=1,\ldots, k+1, $$

are positive and invertible. Thus, the concavity of $\mathcal{P}_{F}$ implies that

$$\begin{aligned} \mathcal{P}_{F}(\lambda S_{1},\ldots, \lambda S_{k+1}) \geq& \lambda\mathcal{P}_{F}(T_{1}, \ldots, T_{k+1})+(1-\lambda )\mathcal{P}_{F}(W_{1}, \ldots, W_{k+1}) \\ \geq& \lambda\mathcal{P}_{F}(T_{1},\ldots, T_{k+1}) . \end{aligned}$$

For $\lambda\rightarrow1$, by continuity, we get

$$\mathcal{P}_{F}( S_{1},\ldots, S_{k+1})\geq \mathcal{P}_{F}(T_{1},\ldots, T_{k+1}). $$

Generally, choose $0<\nu<1$ such that

$$\nu T_{i}< T_{i}\leq S_{i}, \quad i=1,\ldots,k+1. $$

Then we have

$$\mathcal{P}_{F}(\mu T_{1},\ldots, \mu T_{k+1}) \leq\mathcal {P}_{F}(S_{1},\ldots, S_{k+1}). $$

Letting $\nu\rightarrow1$, we get the conclusion. □

### Theorem 4.2

*Suppose that* $F: \mathcal{D}^{k}_{+}\rightarrow\mathfrak {B}(\mathcal{H})_{\mathrm{sa}}$ *is a regular*, *concave*, *and positively homogeneous*. *Then the perspective function* $\mathcal{P}_{F}$ *satisfies the property of congruence invariance*:

4.1 $$\begin{aligned} \mathcal{P}_{F}\bigl(W^{*}T_{1}W,\ldots,W^{*}T_{k+1}W \bigr)= W^{*}\mathcal{P}_{F}(T_{1},\ldots, T_{k+1})W \end{aligned}$$

*for any invertible operator* *W* *on* $\mathcal{H}$.

### Proof

It follows from Theorem 3.2 of [7] that the perspective function $\mathcal{P}_{F}$ is concave. Moreover, since *F* is positively homogeneous, it is easy to prove that $\mathcal{P}_{F}$ is also positively homogeneous. Hence, by Proposition 2.3 in [7], we get the conclusion. □

Let

$$\beta=(\beta_{1},\ldots, \beta_{k})\in[0,1]^{k} $$

with $\sum_{i=1}^{k}\beta_{i}=1$, and let $\alpha_{k+1}\in[0,1]$ and

$$\alpha= (\alpha_{1}, \ldots, \alpha_{k}, \alpha_{k+1} )= \bigl(\beta _{1}(1-\alpha_{k+1}), \ldots, \beta_{k}(1-\alpha_{k+1}), \alpha_{k+1} \bigr). $$

Then, simulated by the significant work of Hansen [7], we construct a sequence of weighted multivariate geometric means $G^{\alpha }_{1}, G^{\alpha}_{2},\ldots$ as follows.

(i) Let $G^{\alpha}_{1}(T)=T$ for each positive definite invertible operator *T*.

(ii) To each weighted geometric mean $G_{k}^{\beta}$ of *k* variables we associate an auxiliary mapping $A_{k}: \mathcal {D}^{k}_{+}\rightarrow\mathfrak{B}(\mathcal{H})$ such that $A_{k}$ is regular and concave, and

$$A_{k}(T_{1},\ldots,T_{k})=G_{k}^{\beta}(T_{1}, \ldots,T_{k})^{(1-\alpha _{k+1})}=\bigl(T_{1}^{\beta_{1}}\cdots T_{k}^{\beta_{k}}\bigr)^{(1-\alpha_{k+1})} $$

for positive $T_{1},\ldots, T_{k}$, where *β* is the weight associated to $T_{1},\ldots, T_{k}$.

(iii) Define the geometric mean $G_{k+1}^{\alpha}: \mathcal {D}^{k+1}_{+}\rightarrow\mathfrak{B}(\mathcal{H})$ of $k+1$ variables as

$$G_{k+1}^{\alpha}(T_{1},\ldots, T_{k+1})= \mathcal{P}_{A_{k}}(T_{1},\ldots, T_{k+1}), $$

where

$$\mathcal{P}_{A_{k}}(T_{1},\ldots, T_{k}, T_{k+1})=T_{k+1}^{1/2}{A_{k}} \bigl(T_{k+1}^{-1/2}T_{1}T_{k+1}^{-1/2}, \ldots ,T_{k+1}^{-1/2}T_{k}T_{k+1}^{-1/2} \bigr)T_{k+1}^{1/2}. $$

Particularly, the geometric means of two variables

$$\begin{aligned} G_{2}^{\alpha}(T_{1},T_{2})=T_{2}^{\frac{1}{2}} \bigl(T_{2}^{-\frac {1}{2}}T_{1}T_{2}^{-\frac{1}{2}} \bigr)^{\alpha_{1}}T_{2}^{\frac{1}{2}} \end{aligned}$$

coincide with the weighted geometric means of two variables $T_{1}\sharp _{\alpha_{1}}T_{2}$ in the sense of Kubo and Ando [6], where $\alpha=(\alpha_{1},\alpha_{2})$ satisfy $\alpha_{1}+\alpha_{2}=1$.

In the above procedure, $\alpha_{i} $ is determined by $\beta_{i} $ and $\alpha_{k+1}$ in the following sense:

$$\alpha_{i}=\beta_{i} (1-\alpha_{k+1}) \quad(i=1, \ldots,k). $$

Conversely for fixed $\alpha=(\alpha_{1},\ldots, \alpha_{k+1})$, we can set

$$\beta_{i}=\frac{\alpha_{i}}{1-\alpha_{k+1}},\quad i=1,2,\ldots,k, \alpha_{k+1}\neq1, $$

and hence trace back to the case of $k=1$. Therefore for fixed weight we can define the corresponding weighted geometric mean.

### Theorem 4.3

*The means* $G_{k}^{\alpha}: \mathcal{D}^{k}_{+}\rightarrow \mathfrak{B}(\mathcal{H})^{+}$ *constructed as above are regular*, *positively homogeneous*, *concave*, *and they satisfy*

4.2 $$\begin{aligned} G_{k+1}^{\alpha}(T_{1}, \ldots,T_{k},1)=G_{k}^{\beta}(\mathbb{T})^{(1-\alpha_{k+1})} \end{aligned}$$

*for* $\mathbb{T}=(T_{1},\ldots T_{k})\in \mathcal{D}^{k}_{+}$.

### Proof

By the definition of $G_{k}^{\alpha}$, we know that $G_{k}^{\alpha}$ for each $k=2,3,\ldots$ is the perspective of a regular positively homogeneous map. Therefore, $G_{k}^{\alpha}$ are regular and positively homogeneous. Moreover, since $G_{k+1}^{\alpha}$ is the perspective of $(G_{k}^{\beta})^{1-\alpha_{k+1}}$, we see that (4.2) holds.

Next, we prove that $G_{k}^{\alpha}$ is concave. Clearly, $G_{1}^{\alpha}$ is concave. Assume that $G^{\beta}_{k}$ is concave for some *k* and the corresponding weight *β*. For $\alpha_{k+1}\in[0,1]$, the map $x\rightarrow x^{1-\alpha_{k+1}}$ is operator monotone (increasing) and operator concave. Then we have

$$\begin{aligned} \bigl(G_{k}^{\beta}\bigl(\lambda\mathbb{T}+(1-\lambda ) \mathbb{S}\bigr) \bigr)^{(1-\alpha_{k+1})} \geq& \bigl( \lambda\bigl(G_{k}^{\beta} \mathbb{T}\bigr)+(1-\lambda) \bigl(G_{k}^{\beta }\mathbb{S}\bigr) \bigr)^{(1-\alpha_{k+1})} \\ \geq& \lambda \bigl(G_{k}^{\beta}\mathbb{T} \bigr)^{(1-\alpha _{k+1})}+(1-\lambda) \bigl(G_{k}^{\beta}\mathbb{S} \bigr)^{(1-\alpha_{k+1})}, \end{aligned}$$

where $\mathbb{T}=(T_{1},\ldots, T_{k})$, $\mathbb{S}=(S_{1},\ldots, S_{k})$. So the auxiliary mapping

$$A_{k}(T_{1},\ldots, T_{k})=G_{k}^{\beta}(T_{1}, \ldots, T_{k})^{1-\alpha_{k+1}} $$

is concave. Then by Theorem 3.2 in [7] we see that its perspective $G_{k+1}^{\alpha}$ is also concave. By induction, we know that $G^{\alpha}_{k}$ is concave for all $k=1,2,\ldots$ . □

### Remark 4.4

A similar analysis to Theorem 4.3 in [7] shows that the above conditions uniquely determine the Geometric means $G_{k}^{\alpha}$ for $k=1,2,\ldots$ by setting $G_{1}^{\alpha}(T)=T$.

### Theorem 4.5

*Set* $\mathbb{T}=(T_{1},\ldots, T_{k})\in\mathcal{D}^{k}_{+}$. *The means* $G_{k}^{\alpha}$ *constructed as above have the following properties*:

1. (consistency with scalars) $G_{k}^{\alpha}(\mathbb {T})=T_{1}^{\alpha_{1}}\cdots T_{k}^{\alpha_{k}}$ *if the* $T_{i}$ *’s commute*;
2. (joint homogeneity) $G_{k}^{\alpha}(t_{1} T_{1},\ldots ,t_{k} T_{k}) =t_{1}^{\alpha_{1}}\cdots t_{k}^{\alpha_{k}}G_{k}^{\alpha}(\mathbb {T})$ *for* $t_{i}>0 $;
3. (monotonicity) *if* $B_{i}\leq T_{i}$ *for all* $1\leq i\leq k$, *then* $G_{k}^{\alpha}(\mathbb{B}) \leq G_{k}^{\alpha}(\mathbb{T})$;
4. (congruence invariance) $G_{k}^{\alpha}(W^{*} T_{1} W,\ldots, W^{*} T_{k} W)=W^{*}G_{k}^{\alpha}(\mathbb{T}) W$ *for any invertible operator* *W* *on* $\mathcal{H}$;
5. (self-duality) $G_{k}^{\alpha}(\mathbb {T}^{-1})=G_{k}^{\alpha}(\mathbb{T})^{-1}$;
6. (A-G-H weighted mean inequalities) $(\sum_{i=1}^{k} \alpha_{i} T_{i}^{-1})^{-1}\leq G_{k}^{\alpha}(\mathbb{T})\leq\sum_{i=1}^{k} \alpha_{i} T_{i}$;
7. (determinant identity) $\det G_{k}^{\alpha }(\mathbb{T})=\Pi_{i=1}^{k}(\det T_{i})^{\alpha_{i}}$.

### Proof

If $T_{1}$ and $T_{2}$ commute, then

$$G_{2}^{\alpha}(T_{1},T_{2})=T_{2}^{\frac{1}{2}} \bigl(T_{2}^{-\frac{1}{2}}T_{1}T_{2}^{-\frac {1}{2}} \bigr)^{\alpha_{1}}T_{2}^{\frac{1}{2}}=T_{1}^{\alpha_{1}}T_{2}^{\alpha_{2}}. $$

Hence, (P1) holds for $k=1,2$. Now assume that (P1) holds for some $k>2$. Since

$$\begin{aligned} G_{k+1}^{\alpha}(T_{1},\ldots,T_{k+1}) =&\mathcal{P}_{F_{k}}(T_{1},\ldots,T_{k+1}) \\ =&T_{k+1}^{\frac{1}{2}}F_{k} \bigl(T_{k+1}^{-\frac{1}{2}}T_{1} T_{k+1}^{-\frac{1}{2}},\ldots, T_{k+1}^{-\frac{1}{2}}T_{k} T_{k+1}^{-\frac {1}{2}} \bigr)T_{k+1}^{\frac{1}{2}} \\ =&T_{k+1}^{\frac{1}{2}}G^{\beta}_{k} \bigl(T_{k+1}^{-\frac{1}{2}}T_{1} T_{k+1}^{-\frac{1}{2}}, \ldots, T_{k+1}^{-\frac{1}{2}}T_{k} T_{k+1}^{-\frac {1}{2}}\bigr)^{(1-\alpha_{k+1})}T_{k+1}^{\frac{1}{2}} \\ =& T_{k+1}^{\frac{1}{2}} \bigl( \bigl(T_{k+1}^{-\frac{1}{2}}T_{1} T_{k+1}^{-\frac{1}{2}} \bigr)^{\beta_{1}}\cdots \bigl(T_{k+1}^{-\frac {1}{2}}T_{k} T_{k+1}^{-\frac{1}{2}} \bigr)^{\beta_{k}} \bigr)^{(1-\alpha _{k+1})}T_{k+1}^{\frac{1}{2}} \\ =& T_{1}^{\beta_{1}(1-\alpha_{k+1})} \cdots T_{k}^{\beta_{k}(1-\alpha _{k+1})}T_{k+1}^{1-(1-\alpha_{k+1})} \\ =& T_{1}^{\alpha_{1}} \cdots T_{k}^{\alpha_{k}}T_{k+1}^{\alpha_{k+1}}, \end{aligned}$$

we see that (P1) also holds for $k+1$. By induction, we know that (P1) holds for $k=1,2,\ldots$ .

It is easy to verify (P2) holds for $k=1$ and $k=2$. Assume that (P2) holds for some $k>2$. Then we have

$$\begin{aligned} &{G_{k+1}^{\alpha}(t_{1}T_{1},\ldots, t_{k}T_{k},t_{k+1}T_{k+1})} \\ &{\quad = t_{k+1}T_{k+1}^{\frac{1}{2}}F_{k} \bigl(t_{1}t_{k+1}^{-1}T_{k+1}^{-\frac {1}{2}}T_{1}T_{k+1}^{-\frac{1}{2}}, \ldots,t_{k}t_{k+1}^{-1}T_{k+1}^{-\frac {1}{2}}T_{k}T_{k+1}^{-\frac{1}{2}} \bigr)T_{k+1}^{\frac{1}{2}}} \\ &{\quad = t_{k+1}T_{k+1}^{\frac{1}{2}}G_{k}^{\beta} \bigl(t_{1}t_{k+1}^{-1}T_{k+1}^{-\frac{1}{2}}T_{1}T_{k+1}^{-\frac{1}{2}}, \ldots ,t_{k}t_{k+1}^{-1}T_{k+1}^{-\frac{1}{2}}T_{k}T_{k+1}^{-\frac {1}{2}} \bigr)^{(1-\alpha_{k+1})}T_{k+1}^{\frac{1}{2}} .} \end{aligned}$$

By induction, we get

$$\begin{aligned} &{G_{k+1}^{\alpha}(t_{1}T_{1},\ldots, t_{k}T_{k},t_{k+1}T_{k+1})} \\ &{\quad =t_{k+1}\bigl(t_{k+1}^{-1}t_{1}^{\beta_{1}} \cdots t_{k}^{\beta_{k}}\bigr)^{(1-\alpha _{k+1})}G_{k+1}^{\alpha}(T_{1}, \ldots, T_{k},T_{k+1})} \\ &{\quad = t_{1}^{\alpha_{1}}\cdots t_{k}^{\alpha_{k}}t_{k+1}^{\alpha _{k+1}}G_{k+1}^{\alpha}(T_{1}, \ldots, T_{k},T_{k+1}) .} \end{aligned}$$

Hence (P2) is true.

(P3) and (P4) follow from Theorems 4.1 and 4.2.

Clearly, (P5) is true for $k=1$ and $k=2$. Assume that (P5) is true for some $k>2$. Then we have

$$\begin{aligned} &{ G_{k+1}^{\alpha}\bigl(T_{1}^{-1},\ldots, T_{k}^{-1},T_{k+1}^{-1}\bigr)} \\ &{\quad = T_{k+1}^{-\frac{1}{2}}F_{k}\bigl(T_{k+1}^{\frac {1}{2}}T_{1}^{-1}T_{k+1}^{\frac{1}{2}}, \ldots,T_{k+1}^{\frac {1}{2}}T_{k}^{-1}T_{k+1}^{\frac{1}{2}} \bigr)T_{k+1}^{-\frac{1}{2}}} \\ &{\quad =T_{k+1}^{-\frac{1}{2}}G_{k}^{\beta} \bigl(T_{k+1}^{\frac {1}{2}}T_{1}^{-1}T_{k+1}^{\frac{1}{2}}, \ldots,T_{k+1}^{\frac {1}{2}}T_{k}^{-1}T_{k+1}^{\frac{1}{2}} \bigr)^{(1-\alpha_{k+1})}T_{k+1}^{-\frac{1}{2}} .} \end{aligned}$$

By induction, we get

$$\begin{aligned} &{G_{k+1}^{\alpha}\bigl(T_{1}^{-1},\ldots, T_{k}^{-1},T_{k+1}^{-1}\bigr)} \\ &{\quad = T_{k+1}^{-\frac{1}{2}}G_{k}^{\beta} \bigl(T_{k+1}^{-\frac {1}{2}}T_{1}T_{k+1}^{-\frac{1}{2}}, \ldots,T_{k+1}^{-\frac {1}{2}}T_{k}T_{k+1}^{-\frac{1}{2}} \bigr)^{-(1-\alpha_{k+1})}T_{k+1}^{-\frac{1}{2}}} \\ &{\quad =\bigl(T_{k+1}^{\frac{1}{2}}G_{k}^{\beta} \bigl(T_{k+1}^{-\frac {1}{2}}T_{1}T_{k+1}^{-\frac{1}{2}}, \ldots,T_{k+1}^{-\frac {1}{2}}T_{k}T_{k+1}^{-\frac{1}{2}} \bigr)^{(1-\alpha_{k+1})}T_{k+1}^{\frac {1}{2}}\bigr)^{-1}} \\ &{\quad = G_{k+1}^{\alpha}(T_{1},\ldots, T_{k},T_{k+1})^{-1},} \end{aligned}$$

which verifies (P5).

The A-G-H weighted mean inequality, i.e. the arithmetic-geometric-harmonic weighted mean inequality reads

$$\Biggl(\sum_{i=1}^{k} \alpha_{i} T_{i}^{-1}\Biggr)^{-1}\leq G_{k}^{\alpha}(T_{1}, \ldots ,T_{k})\leq\sum_{i=1}^{k} \alpha_{i} T_{i} $$

for arbitrary $(T_{1},\ldots,T_{k})\in\mathcal{D}^{k}_{+}$. Firstly, we show the second inequality. It is easy to see the second inequality holds for $k=1$. Assume the inequality holds for some *k*. Then, by virtue of

$$X^{p}\leq1+p(X-1) $$

for $X\in\mathcal{D}^{1}$ (the set of positive operators) and $p\in [0,1]$, we obtain

$$\begin{aligned} F_{k}(T_{1},\ldots, T_{k}) =& G_{k}^{\beta}(T_{1}, \ldots, T_{k})^{(1-\alpha_{k+1})} \\ \leq& 1+(1-\alpha_{k+1}) \bigl(G_{k}^{\beta}(T_{1}, \ldots, T_{k})-1\bigr) \\ \leq& 1+(1-\alpha_{k+1}) \Biggl(\sum_{i=1}^{k} \beta_{i} T_{i}-1 \Biggr) \\ =&\sum_{i=1}^{k} (1-\alpha_{k+1}) \beta_{i} T_{i}+\alpha_{k+1} =\sum_{i=1}^{k} \alpha_{i} T_{i}+\alpha_{k+1}. \end{aligned}$$

Now taking perspective, we have

$$\begin{aligned} G_{k+1}^{\alpha}(T_{1},\ldots, T_{k}, T_{k+1}) =&\mathcal {P}_{F_{k}}(T_{1},\ldots, T_{k}, T_{k+1}) \\ =&T_{k+1}^{\frac{1}{2}}F_{k}\bigl(T_{k+1}^{-\frac{1}{2}}T_{1}T_{k+1}^{-\frac {1}{2}}, \ldots, T_{k+1}^{-\frac{1}{2}}T_{k}T_{k+1}^{-\frac {1}{2}} \bigr)T_{k+1}^{\frac{1}{2}} \\ \leq& T_{k+1}^{\frac{1}{2}} \Biggl(\sum_{i=1}^{k} \alpha_{i} T_{k+1}^{-\frac {1}{2}}T_{i}T_{k+1}^{-\frac{1}{2}}+ \alpha_{k+1} \Biggr) T_{k+1}^{\frac {1}{2}} \\ = &\sum_{i=1}^{k+1} \alpha_{i} T_{i}. \end{aligned}$$

By induction, the second inequality is proved. Next, it follows from the second inequality that

$$G_{k}^{\alpha}\bigl(T_{1}^{-1}, \ldots,T_{k}^{-1}\bigr)\leq\sum_{i=1}^{k} \alpha_{i} T_{i}^{-1}. $$

By inversion and using the self-duality (P7) of the weighted geometric mean, we get

$$\Biggl(\sum_{i=1}^{k} \alpha_{i} T_{i}^{-1}\Biggr)^{-1}\leq G_{k}^{\alpha} \bigl(T_{1}^{-1},\ldots ,T_{k}^{-1} \bigr)^{-1}=G_{k}^{\alpha}(T_{1}, \ldots,T_{k}). $$

Hence the property (P6) holds.

For $T\in\mathcal{D}^{1}$ and $p\in\mathbb{R}$, we have $\det T^{p}=(\det T)^{p}$ due to $\det T=\exp(\textrm{Tr}\log T)$. For $k=1$ and $k=2$, (P7) is obviously correct. Assume that (P7) holds for some $k>2$. Then, using

$$\begin{aligned} G_{k+1}^{\alpha}(T_{1},\ldots, T_{k}, T_{k+1}) =T_{k+1}^{\frac{1}{2}}G_{k}^{\beta} \bigl(T_{k+1}^{-\frac {1}{2}}T_{1}T_{k+1}^{-\frac{1}{2}}, \ldots,T_{k+1}^{-\frac {1}{2}}T_{k}T_{k+1}^{-\frac{1}{2}} \bigr)^{(1-\alpha_{k+1})}T_{k+1}^{\frac {1}{2}} , \end{aligned}$$

we infer that

$$\begin{aligned} \det G_{k+1}^{\alpha}(T_{1},\ldots, T_{k}, T_{k+1}) =& \det T_{k+1}\bigl(\det T_{k+1}^{-\beta_{1}}\det T_{1}^{\beta_{1}}\cdots\det T_{k+1}^{-\beta_{k}}\det T_{k}^{\beta_{k}}\bigr)^{(1-\alpha_{k+1})} \\ =&\prod_{i=1}^{k+1}(\det T_{i})^{\alpha_{i}}, \end{aligned}$$

which means that (P7) is true. □
